# The pregnancy microbiome and preterm birth

**DOI:** 10.1007/s00281-020-00817-w

**Published:** 2020-08-14

**Authors:** Erna Bayar, Phillip R. Bennett, Denise Chan, Lynne Sykes, David A. MacIntyre

**Affiliations:** 1 Imperial College Parturition Research Group, Institute for Reproductive and Developmental Biology, Imperial College London, Du Cane Road, London, W12 0HS UK; 2 March of Dimes European Preterm Birth Research Centre, Imperial College London, London, UK

**Keywords:** Preterm birth, Pregnancy, Vaginal microbiome, Lactobacillus

## Abstract

Preterm birth is a global health concern and continues to contribute to substantial neonatal morbidity and mortality despite advances in obstetric and neonatal care. The underlying aetiology is multi-factorial and remains incompletely understood. In this review, the complex interplay between the vaginal microbiome in pregnancy and its association with preterm birth is discussed in depth. Advances in the study of bacteriology and an improved understanding of the human microbiome have seen an improved awareness of the vaginal microbiota in both health and in disease.

## Introduction

Preterm birth (PTB) is a multi-aetiological disease state that causes almost 1 million deaths each year making it the primary cause of mortality in children under 5 years of age worldwide [1]. Infection is thought to contribute to at least one third of these cases [2, 3]. Whilst systemic maternal infection and colonisation of the lower reproductive tract by known pathogens such as *Trichomonas vaginalis* and *Chlamydia trachomatis* have long been associated with increased risk of PTB, recent applications of molecular-based profiling methods have provided new insights into the role that microbe-host interactions in pregnancy play in shaping PTB risk [4, 5]. In this review, we examine the current evidence linking maternal microbiota composition and host response to high-risk PTB phenotypes.

## Culture- and molecular-based profiling of microbial communities

Bacteriology in the late nineteenth and twentieth centuries was largely limited to the investigation of microorganisms that were easily amenable to isolation and culture outside of the human body. However, advances in microscopy quickly led to the realisation that human body niches were colonised by many different microbial morphotypes of a complexity that culture-based methods alone failed to capture. In the twenty-first century, the application of culture-free, molecular-based approaches such as high-throughput DNA sequencing techniques has provided a step change in our ability to rapidly and comprehensively characterise polymicrobial communities and has led to a greater appreciation of the vast numbers of bacteria, viruses, fungi and archaea that inhabit the human body [6]. The sum total of microorganisms present in a defined community is referred as the ‘microbiota’, whereas the ‘microbiome’ refers to the entire habitat, including the microbiota, their genomes and the surrounding environmental conditions [7].

Broadly, two sequencing-based strategies are commonly used to study the microbiota; ‘metagenomics’ and ‘metataxonomics’. The metagenome refers to the collection of genomes and genes from the members of a microbiota community and is obtained through shotgun sequencing of all of DNA present in a given sample [7]. Metataxonomics involves amplification and sequencing of specific, often short-length regions of microbial taxonomic marker genes. For bacteria, the 16S ribosomal RNA gene (16S rRNA) is most commonly targeted, whereas the internal transcribed spacer (ITS) region is often used for characterisation of fungi. The bacterial 16S rRNA gene is composed of highly conserved regions and nine ‘hyper-variable’ regions (V1-V9). PCR primers designed to bind to the conserved regions permit amplification of the variable regions, which are generally diverse and distinctive thus facilitating classification of bacterial taxonomy to species, and in some cases strain level, by mapping the resulting sequences to existing 16S rRNA gene databases [8]. Accurate identification of bacterial species using metataxonomics is therefore prone to bias depending on the choice of amplification region, primer design and even the database that the sequence reads are mapped to. For example, the ‘conserved’ regions of the 16S rRNA gene are not fully conserved so PCR primers may selectively bind to some but not all bacterial DNA in the sample. Moreover, ‘hyper-variable’ regions will not always provide sufficient variability to distinguish between species, let alone strains of bacteria. Such issues are important to consider when examining and interpreting results of microbiota studies particular in the context of studies focussed on the reproductive tract microbiota [9]. For example, the targeting of the V4 hyper-variable region, which is commonly used to examine the gut microbiota, gives poor resolution of *Lactobacillus* species, a keystone commensal species of the cervicovaginal niche. Conversely, whilst the VI–V2 region differentiates *Lactobacillus* species well, universal primers may fail to amplify the important vaginal pathobiont, *Gardnerella vaginalis*, due to it having a less conserved sequence in the pre-V1 conserved region. Such difficulties can be overcome by careful design and use of degenerate PCR primers; however, it is important to recognise that rare, but physiologically important, taxa may be missed through selective amplification of high-abundance species.

In contrast to metataxonomic profiling, metagenomics approaches provide strain-level resolution through shotgun sequencing and bioinformatic assembly of microbial genomes, which can then be mapped and annotated using reference databases. Metagenomic sequencing avoids PCR bias and captures information of the collective genomes from all then constituents of the microbiome kingdom. The resulting information can be leveraged to provide insight into potential metabolic function in additional to compositional structure of the community under investigation. Disadvantages have historically included higher costs, greater computation burden and inability to detect rare or low-abundance taxa in low-biomass samples that are overwhelmed by DNA from host and highly abundant commensals. These issues are increasingly less of a concern as sequencing costs continue to fall and new methods for sample preparation and bioinformatics approaches are developed [10].

## The vaginal microbiota in health and disease

The vaginal microbiota plays a key role in female genital tract health and disease [11]. Throughout a woman’s lifespan, the composition of the vaginal microbiota is shaped by both intrinsic and extrinsic factors. Prior to the menarche low levels of circulating oestrogen associate with a high-diversity microbial structure consisting of aerobic, anaerobic and enteric bacteria [12]. Menarche is associated with increasing circulating oestrogen levels which promote proliferation of vaginal epithelial cells and glycogen deposition, the breakdown products of which can be preferentially utilised by *Lactobacillus* spp. as a primary carbon source, favouring their dominance and leading to a fall in pH [13]. This acidic environment, enriched by bacteriocins and other antimicrobial compounds produced by *Lactobacillus* spp., results in a hostile mucosal microenvironment that they have evolved and adapted to thrive in, which is hostile to many other microbial species. In this way, *Lactobacillus* spp. are considered to be keystone members of the vaginal microbiome during a woman’s reproductive years and are often associated with states of health and protection against bacterial vaginosis (BV), pelvic inflammatory disease, candidiasis, sexually transmitted infections, HIV, HSV-2 and carcinogenic human papilloma virus [14–19].

The first application of next-generation sequencing–based approaches to characterise the bacterial component of the vaginal microbiota was published by Ravel et al. [15]. Using hierarchical clustering of relative abundance data derived from metataxonomic profiling of vaginal samples collected from asymptomatic women, they described five distinct vaginal community state types (CST). Four of these state types were dominated by *Lactobacillus* spp. (*L. crispatus*, *L. gasseri*, *L. iners* or *L. jensenii*, designated CST I, II, II and V respectively). The remaining CST, CST IV, was *Lactobacillus* spp. depleted and characterised by a highly diverse, polymicrobial community that compositionally resembled that of women with a clinical diagnosis of bacterial vaginosis. This schema was later modified to account for differences within the *Lactobacillus* spp. deplete, high-diversity groups with CST IV-A, characterised by enrichment for *Peptoniphilus* and *Prevotella* species, and CST IV-B, characterised by a higher relative abundance of *Atopobium* and *Gardnerella* species and other taxa previously associated with bacterial vaginosis. Whilst other statistical methodologies have since been used to classify vaginal microbiota profiles (also referred to as ‘vagitypes’ or ‘vaginal microbial communities’), their compositional characteristics remain relatively consistent despite rarer vaginal microbial community types increasingly being reported (e.g. Bifidobacteria-dominated profiles).

Longitudinal profiling of vaginal microbiota has shown that whilst some women maintain a level of compositional stability throughout the menstrual cycle and at the time of menstruation, falling oestrogen and progesterone, endometrial shedding and vaginal bleeding lead to dynamic shifts in composition, often characterised by reduced *Lactobacillus* sp. relative abundance, which recovers during the follicular phase of the cycle [20]. Pregnancy brings endocrine stability, a substantial increase in circulating oestrogen concentrations and an end to menstrual bleeding which favours *Lactobacillus* sp. dominance of the vaginal microbiota [21]. Several cohort studies have shown that healthy pregnant women with normal outcomes generally maintain a vaginal microbiota dominated by *Lactobacillus* spp. throughout the entire pregnancy. The shift towards *Lactobacillus* sp. dominance appears to occur early in pregnancy and is most dramatically observed in women of African ancestry. Following delivery, maternal oestrogen levels fall to menopausal levels, which, coupled with the passage of lochia, changes the vaginal environment resulting in decreased *Lactobacillus* spp. and a shift towards *Lactobacillus* spp. [21] depleted community structures that have been reported to persist for up to one year postpartum [22].

## The vaginal microbiome and preterm birth

Intrauterine infection leading to PTB is widely hypothesised to be secondary to pathogen ascension from the vagina [23, 24]. In humans, this notion is supported by the similarity observed between bacterial species found in the placenta and fetal membranes (amnion and chorion) of PTB cases and those found in the vagina [25, 26]. The fact that histological chorioamnionitis is most frequently observed at the site of membrane rupture in the lower part of the uterus close to the cervix [27] and in twin pregnancies, where histological chorioamnionitis and microbial invasion of the amniotic cavity are most commonly observed in the presenting and first born twin further supports the notion [25, 28]. The process of ascending vaginal infection leading to PTB can also be readily replicated in a number of animal models [29–34].

Given this evidence, characterisation of the vaginal microbiota in women with PTB or preterm prelabour rupture of the membranes (PPROM) has gained increasing attention. As commonly occurs in burgeoning research areas, the earliest of these studies were limited by a lack of power and a lack of consideration of underlying aetiology, which lead to inconsistent reports of relationships, or lack thereof, between vaginal microbiota and PTB. In the earliest of such molecular-based studies of vaginal microbiota in PTB, Hyman et al. reported that bacterial diversity was greater in Caucasians with PTB but that species diversity was generally higher among African Americans [35]. In contrast, Romero et al. found no differences in bacterial taxa abundance or community state types between women delivering preterm or term; however, this cohort was almost entirely African American [36]. The concept that ethnicity is a strong determinant of the vaginal microbiome and its effect upon PTB rates was further developed in two studies from the Relman group in Stanford. The first in a mostly White population correlated *Lactobacillus*-depleted vaginal community state types with reduced gestational age at delivery and showed that risk for PTB was greater in women with high abundances of *Gardnerella* or *Ureaplasma* [22]. The subsequent study compared two populations, White women in California and Black women in Alabama, and showed that whilst *Lactobacillus* depletion and greater abundance of *Gardnerella* were more common in Black women, it represented a risk factor for PTB only in White women. *Lactobacillus crispatus* was found to be protective against PTB in both groups [37].

A study on a small group of nulliparous African American women reported a trend between spontaneous PTB and lower vaginal bacterial diversity, although this was not statistically significant [38]. Similarly in a cohort of Black women, abundance of specific *Lactobacillus* species did not correlate to risk or protection from PTB, but a decrease in vaginal diversity was associated with PTB in African American women [39]. Recently, differences between bacterial taxa and PTB between African American and non-African American women have been explored [40]. Again, whilst more African Americans had reduced abundance of *Lactobacillus* species, this was only a significant risk factor for PTB in White women. This study also reported the identification of specific bacterial taxa that were significantly associated with spontaneous PTB, with a stronger effect observed in African American women. However, in this cohort, higher β-defensin-2 lowered the risk of spontaneous PTB in Black women.

As part of the integrative Human Microbiome Project, Fettweiss et al. compared the vaginal microbiome and cytokine profile between 45 women delivering preterm and 90 women delivering term of African descent, matched for age and income. Metaxonomic, metagonomic and cytokine analyses showed those delivering preterm had significantly lower levels of *Lactobacillus crispatus*, higher levels of BVAB1, *Sneathia amnii*, TM7-HI and a group of *Prevtolla* species [41]. Women that delivered term were more likely to have *Lactobacillus crispatus* and decreased prevalence of *A. vaginae* and *G. vaginalis*. PTB was also associated with a vaginal cytokine profile richer in pro-inflammatory cytokines, including eotaxin, IL-1β, IL-6 and MIP-1β.

European studies have tended to comprise mostly of White women. In a study of women of European, Middle Eastern or Asiatic origin, an increased risk of PTB associated with *Lactobacillus iners* and a protective effect for *Lactobacillus crispatus* dominance was shown [42]. Similarly, we previously showed that in a UK population, *Lactobacillus iners* dominance at 16 weeks was significantly associated with both a short cervix and PTB before 34 weeks [43]. In contrast, *Lactobacillus crispatus* dominance was highly predictive of term birth. In this study, neither cervical shortening nor PTB were associated with high-diversity community compositions; however, the prevalence of *Lactobacillus* depletion in our cohort was relatively low. The concept that the vaginal microbiota may be more important in relation to early preterm birth is supported by Canadian data from predominantly White European women which showed that the predominance of microbial profiles dominated by *Lactobacillus gasseri*/*Lactobacillus johnsonii*, *Lactobacillus crispatus*/*Lactobacillus acidophilus*, *Lactobacillus iners*/*Ralstonia solanacearum* or *Bifidobacterium longum*/*Bifidobacterium breve* is associated with a decreased risk of PTB before 34 weeks of gestation [44]. High-diversity compositions and the presence of BV-associated bacteria (e.g. *G. vaginalis*, *A. vaginae* and *Veillonellaceae bacterium*) were linked to an increased risk of early PTB.

Approximately 30–40% of PTB cases are preceded by PPROM [45]. Rupture of the fetal membranes provides an entry point for ascending microbes, but infection may be both a cause and a consequence of PPROM. Pathogenic vaginal bacteria may ascend and trigger inflammatory cascades, leading to remodelling of the cervix and fetal membranes. Following PPROM, ascending pathogens contribute to the development of chorioamnionitis and funisitis [46–48].

Four studies have examined the vaginal microbiota at the time of PPROM. These show that up to half of women who present with PPROM have a vaginal microbiota characterised by intermediate or low *Lactobacillus* sp. dominance and high diversity [49–52]. Our study found that in individual case samples sampled both before and after PPROM, about half of those with *Lactobacillus* sp. dominance prior to PPROM became dysbiotic post rupture, and that treatment with erythromycin further exacerbates vaginal dysbiosis, characterised by *Lactobacillus* sp. depletion and enrichment for potentially pathogenic bacteria [52]. In this cohort, vaginal dysbiosis was a risk factor for both chorioamnionitis and early onset neonatal sepsis. In a later prospective cohort study, we observed reduced *Lactobacillus* sp. abundance and high diversity in a quarter of women prior to PPROM, but in only 3% of women who delivered at term without membrane rupture [53]. PPROM associated with a shift towards higher diversity, predominately occurring during the second trimester, although a vaginal bacterial community dominated by any species other than *Lactobacillus* was associated with subsequent PPROM at all gestational time windows, including during the first trimester.

Progesterone is now commonly offered to women at risk of PTB particularly in the context of second trimester cervical shortening. In vitro studies have shown that progesterone inhibits inflammatory pathways in amnion and myometrium, reducing cytokine and prostaglandin production [54–56]. Therapeutic progesterone may reduce myometrial contractility, prevent cervical remodelling and increase levels of local antimicrobial proteins, so reducing the risk of PTB [54, 57–59]. A small study which randomised women to progesterone or cervical cerclage showed that whilst cerclage leads to increased levels of inflammatory cytokines in cervicovaginal fluid, progesterone has no effect [60]. Progesterone action similarly does not appear to act through modulation of the vaginal microbiota. In non-pregnant women, Borgdorff et al. found that both injectable progestin contraception and combined oral contraception (progestin and oestrogen) do not significantly alter the vaginal microbiota, but may increase the risk of HIV transmission [61–63]. We have shown that administration of progesterone to women with a short cervix in pregnancy does not lead to changes in the abundance of either *Lactobacillus iners* or *Lactobacillus crispatus.* We showed through longitudinal sampling of women receiving progesterone for a short cervix that *L. iners* dominance persisted and was associated with all women who subsequently delivered preterm. It is therefore likely that the mode of action of progesterone is non-microbial [43].

The management of women presenting with cervical shortening and exposed membranes in the second trimester is challenging. Without intervention, delivery usually follows within 2–3 weeks [64, 65]. Insertion of an emergency or rescue cervical cerclage prolongs gestation and improves birth weight and neonatal survival [66, 67]. However, the take home baby rate following rescue cerclage is only 50% and identifying which women would benefit from a rescue cerclage is challenging [65]. Although management remains contentious, many obstetricians would offer ‘rescue’ cervical cerclage in such cases provided there was no clinical evidence of established preterm labour or chorioamnionitis. Data suggests that a bimodal distribution of the gestational age of delivery in women offered rescue cerclage is due to the underlying aetiology of cervical dilation with a subset of women likely to deliver early because of subclinical infection [66]. Consistent with this, we recently reported in a small cohort of women presenting with a dilated cervix and exposed fetal membranes that reduced *Lactobacillus* sp. relative abundance was observed in 40% of cases prior to cerclage compared with 10% of gestational age-matched controls [68]. *Gardnerella vaginalis* was overrepresented in women presenting with symptoms and those in whom rescue cerclage failed to usefully prolong pregnancy. The insertion of a rescue cerclage, which was using inert monofilament material, did not affect the underlying bacterial composition. These findings point towards at least two underlying aetiologies in women presenting with silent cervical dilatation prior to PPROM, one of which is linked to pathogen colonisation of the cervicovaginal niche and poor response to cervical cerclage, and another which includes a high proportion of women with genuine cervical mechanical abnormality, and who may respond well to cerclage.

## Vaginal microbiota and background PTB risk

There exists a small background increase in the rate of PTB in women with untreated cervical intraepithelial neoplasia (CIN), but the principal risk of PTB relates to the degree of tissue destruction caused by a pre-pregnancy history of a large loop excision of the transformation zone of the cervix (LLETZ) or cone biopsy. In particular, there is a ‘depth-dependent’ relationship between depth of excision of cervical tissue and subsequent risk of PTB [69]. We have shown that in non-pregnant women, CIN associates with an increased prevalence of vaginal microbiota characterised by high diversity and low levels of *Lactobacillus* spp. and that increasing severity of CIN is associated with decreasing relative abundance of *Lactobacillus* spp. [70]. A subsequent study of a cohort of adolescent and young women with histologically confirmed, untreated CIN2 lesions showed that women with *Lactobacillus*-dominant vaginal microbiota at baseline are more likely to have regressive disease at 12 months [71]. Current evidence suggests that LLETZ and similar procedures may improve vaginal microbiota composition. Wiik et al. demonstrated a reduction in the relative abundance of non-*Lactobacillus* spp. following LLETZ procedures [72]. Similarly, a follow-up of women 3 months after LLETZ procedures for CINI/II demonstrated a shift towards *Lactobacillus iners* dominance of vaginal communities [73]. This data collectively highlights vaginal dysbiosis in women with CIN or a previous LLETZ as a possible causal factor for increased risk of PTB in these women. Whilst to the best of our knowledge no published studies of the effect of pregnancy upon the vaginal microbiota in women who have had LLETZ have been reported, we have, however, seen excellent outcomes in women with a prior LLETZ who are managed by surveillance of cervical length during pregnancy with targeted cervical cerclage [74]. The association between PTB and local cervical treatment may therefore be microbial as well as mechanical.

## From compositional awareness to mechanistic understanding

The great majority of studies linking the vaginal microbiota have been essentially associative, with little or no focus upon mechanism. A consistent finding across most studies is the protective effect of *Lactobacillus* species, particularly *L. crispatus*. In addition to producing bacteriocins and other antimicrobial compounds, *L. crispatus* produces mainly d-lactate as its primary metabolic end product, which not only acts to maintain a low pH but also has been reported to inhibit activation of extracellular matrix metalloproteinase inducer (EMMPRIN), an activator of MMP-9 and so of collagen degradation [75]. *L. crispatus* also has beneficial immunomodulatory functions and can inhibit adhesion of other bacteria such as *G. vaginalis* to vaginal epithelial cells [76]. In contrast, the role of *L. iners* in the vaginal microbiota is less clear, since it can be detected in normal conditions as well as during vaginal dysbiosis and as mentioned earlier, a number of studies have identified dominance of vaginal microbiota by *L. iners* as a risk factor for preterm labour. A major difference between *L. iners* and *L. crispatus* is genome size. *L. iners* has an unusually small genome (ca. 1 Mbp compared with 3–4 Mbp) resulting in a lack of key functional metabolic genes. As a result, it seems to have evolved a level of tolerance to co-colonise alongside other vaginal bacteria, including potential pathogens, and is associated with a transitionary state of vaginal microbiota composition compared with *L. crispatus*, which is highly exclusionary [77]. *L. iners* dominance of vaginal communities is also associated with activation of EMMPRIN, and mucin-degrading enzymes which disrupts the immune barrier [75]. It may therefore be the case that unlike *L. crispatus*, *L. iners* fails to prevent pathogen colonisation of the vagina during pregnancy and is therefore indirectly ‘causative’ of high-risk vaginal microbiota communities that associate with PTB or that its ability to coexist with pathobionts and pathogens means that it simply acts as a marker.

Several studies have now reported findings suggesting that one of the ways in which high-diversity vaginal microbiota may increase risk of PTB is through untimely activation of inflammatory pathways involved in the initiation of parturition. In one of the first of such studies, we showed that in women with cervical cerclage, the use of a braided suture material induces a persistent shift towards vaginal microbiome dysbiosis and activation of local inflammation accompanied by ultrasound evidence of premature cervical remodelling [78]. Analyses of retrospective data from multiple centres across the UK also showed that braided cerclages associate with a threefold increase in numbers of intrauterine fetal deaths and a doubling in the rate of PTB compared with cerclage with monofilament suture, which had no significant impact upon the vaginal microbiota, cytokine concentrations or cervical changes. This evidence suggests a direct effect of microbiome-activated inflammation on the cervix developing as the pregnancy progresses. That concept is supported by the finding from our prospective studies of PPROM described earlier that in the majority of cases, PPROM is associated with a shift towards higher diversity occurring during the second or early third trimester, relatively close to the time of PPROM [53]. Fetal leukocyte telomere length is reduced at the time of normal term labour and is a marker for oxidative stress and fetal membrane senescence [79]. Fetal leukocyte telomere length is also reduced in PPROM compared with PTB without prior PPROM but is similar to term births, supporting the hypothesis that PPROM may be in part caused by oxidative stress–induced senescence. A range of factors, including infection or inflammation, can lead to oxidative stress. Mechanical stress may also activate sterile inflammation within the fetal membranes, thus providing an attractive hypothesis for a final common pathway to membrane rupture in the context of both abnormal mechanical forces, the context of cervical mechanical damage, polyhydramnios or multiple pregnancy, or vaginal dysbiosis–driven inflammation within the vagina cervix and lower uterine segment [79].

## Evidence for an oral-placental microbiome axis in preterm birth

Haematogenous spread of microbes and subsequent colonisation of the placenta have also been proposed as a potential secondary route of invasion and infection leading to PTB [80]. It has been suggested that the origin of these bacteria is the oral cavity [81] and oral-to-placental transmission of pathogens can been demonstrated in murine models [82, 83]. Further evidence for an oral-placental axis involved in PTB includes the identification of common oral pathogens (e.g. *Fusobacterium nucleatum*, *Dialister* spp., *Prevotella* spp., and *Porphyromonas gingivalis*) in the placenta of women with periodontal disease [84, 85], which is associated with increased risk of PTB [84, 86, 87]. However, interventions that improve periodontal health during pregnancy do not prevent PTB [88–90] and many bacterial species that were historically considered to be limited to the oral cavity are now recognised as being commonly observed members of the vaginal niche.

Regardless, a large amount of research effort over the last decade has been invested into work describing the existence of a placental microbiome that may influence pregnancy outcome. Controversy continues to plague research in this area, much of which appears to stem from definition of terms with some investigators considering that the term ‘microbiome’ should only be applied to microbial communities that have a well-described biological or physiological function (e.g. gut or vaginal microbiome) in host health. For example, the human gut microbiome plays a wide range of essential physiological functions including nutrient absorption, metabolism, maintenance of mucosal integrity and immunomodulation. The human vaginal microbiome plays important roles in protecting the vagina from overgrowth of pathobionts and pathogens, and prevention of ascending pelvic inflammatory disease or chorioamnionitis. These two ‘microbiomes’ clearly have essential physiological functions. Aagaard et al. published the first study suggesting that the placenta contains a genuine microbiome composed of non-pathogenic microbiota [91]. The placental microbiome profiles were most akin to the human oral microbiome, and differences were seen in women with a history of antenatal urinary tract infection in the first trimester, and with PTB. Metagenomic analysis implied a wide range of potential metabolic functions for these organisms. However, the total biomass of organisms suggested to be in the placenta was very low, which is inconstant with an essential metabolic role. Further, some of the microbes identified in the placenta had previously only been reported in environments very dissimilar to the human placenta, such as soda lakes, alkaline and saline habitats, and marine environments. One organism purportedly associated with PTB was *Gloeobacter*, which is a genus of cyanobacteria (also known as blue-green algae), which obtain their energy through oxygenic photosynthesis.

Subsequent studies using a combination of culture-based, metagenomic and metataxonomic analyses have failed to identify a genuine placental microbiome in normal pregnancy. Lauder et al. compared placental samples from healthy deliveries with a matched set of contamination controls, and oral and vaginal samples from the same women [92]. Placental samples and negative controls contained very low and indistinguishable copy numbers of the 16S rRNA gene. Oral and vaginal swab samples had six orders of magnitude higher copy numbers. It was concluded that all of the bacterial DNA amplified from placental samples derived from environmental or reagent contamination and that results diverged radically in association with the kit used to purify the DNA. Similarly, Theis et al. used a combination of cultivation, quantitative real-time PCR, 16S rRNA gene sequencing and metagenomics to study placentas from a cohort of women who had been delivered by Caesarean-section without labour at term [93]. They also concluded that a normal resident microbiota could not be identified in the human placenta and found no difference in microbiota between placental samples and background technical controls. Recently, Goffau et al. performed metagenomic and metataxonomic analysis to determine whether pre-eclampsia, small for gestational age infants or spontaneous PTB were associated with bacterial DNA in the placenta [94]. No bacteria were found in most placental samples, from either complicated or uncomplicated pregnancies. Almost all signals were related either to the acquisition of bacteria during labour and delivery, or to sources of laboratory contamination. The exception was *Streptococcus agalactiae* (group B streptococcus), for which non-contaminant signals were detected in 5% of samples collected. It therefore seems highly unlikely that there is genuine consistent and physiological relevant normal microbiome within the human placenta.

It does seem likely, however, that some cases of spontaneous preterm labour, particularly those associated with chorioamnionitis, harbour detectable bacteria within the placenta. A large recent UK study of 400 placental samples from 256 singleton pregnancies found no convincing evidence for the existence of a reproducible ‘preterm placental microbiome’ but did find evidence of *Mycoplasma* spp. and *Ureaplasma* spp. associated with PTB [95]. A further study from the Aagaard laboratory has shown that specifically, preterm subjects with severe chorioamnionitis have high abundances of *Ureaplasma parvum*, *Fusobacterium nucleatum* and *Streptococcus agalactiae* within the placenta, supporting the idea of either ascending or haematogenous infection [96].

## The gut microbiome

The gastrointestinal tract (GIT) has the largest surface area in the body that is exposed to foreign material consisting of a diverse community of fungi, protozoa, viruses, archaea and bacteria. The most abundant phyla of bacteria that exist in the human GIT are *Firmicutes*, *Bacteroidetes*, *Actinobacteria* and *Proteobacteria* with *Firmicutes* and *Bacteroidetes* occupying 70–90% of the total GIT bacteria [97, 98]. As many as 1000 species have been identified as having critical physiological roles that are essential to the host, including regulation of immune homeostasis. Evidence for this is supported by germ-free mice exhibiting an altered immune phenotype with deficits in the local innate and adaptive immune components [99, 100].

Several studies have sampled the gut microbiota across longitudinal timepoints in pregnancy as gestational age advances, with the majority reporting that the diversity and composition of bacterial communities remain largely stable throughout pregnancy [22, 97, 101]. However, the largest study of 91 women by Koren et al. showed a decrease in α-diversity and an expansion of β-diversity between the first and third trimesters, which was associated with an increase in pro-inflammatory cytokine concentrations in stool [102]. They also describe an increase in the relative abundance of *Proteobacteria* and *Actinobacteria* in the third trimester in 69% and 57% of women respectively. OTUs overrepresented in the third trimester were of the *Enterobacteriaceae* family and *Streptococcus* genus, whereas OTUs overrepresented in the first trimester were the anti-inflammatory butyrate producers *Eubacterium* and *Faecalibacterium*. In addition, transfer of third trimester maternal microbiota to germ-free mice induced a mild inflammatory response, with higher stool IL-1β. It is therefore plausible that microbiome remodelling follows a distinct temporal adaptation in order to allow for gestational age–dependent immunomodulation to facilitate successful pregnancy and timely parturition.

The concept of both local and systemic temporal immune adaptation in healthy pregnancy is well-established. The beneficial anti-inflammatory state allows to accommodate the developing fetus during pregnancy, until a shift towards a pro-inflammatory state occurs in the third trimester to allow for parturition [103]. Given the evidence supporting the role of a systemic immunological clock in the timing of delivery, it is plausible that unfavourable and premature alterations in composition and diversity of the gut microbiota, resulting in systemic inflammation, could play a role in the aetiology of spontaneous preterm labour [104]. In addition, since there are close anatomical links between the lower reproductive tract and the lower intestinal tract, it is also plausible that microbial seeding between the gut and vagina can influence the microbial environment of both sites, leading to aberrant local inflammation.

Dahl et al. examined day 4 postpartum faecal samples from 19 women who delivered preterm and 102 women who delivered at term and revealed a reduction in α-diversity and lower operational taxonomic unit (OTU) abundances of the *Bifidobacterium* and *Streptococcus* genera, and of families in the *Clostridiales* order [105]. *Bifidobacterium* has many anti-inflammatory properties with multiple strains able to inhibit LPS-induced NF-κB activation, IL-8 and COX-2 production in vitro [106, 107]. Therefore, a reduction in *Bifidobacterium* could lead to increased susceptibility to inflammation/infection-induced preterm labour. Shiozaki et al. showed a reduction in OTUs from several species of *Clostridium* and *Bacteroides* in 10 women who delivered preterm compared with 10 women who delivered at term [108]. *Clostridia* spp. are potent inducers of T regulatory cell number and activation [109, 110]. *Bacteroides fragilis* promotes an anti-inflammatory response in the gut by activating IL-10 secreting T-reg cells via polysaccharide A. This suppresses the Toll-like receptor (TLR) 2 signalling pathway and dampens Th17 responses [111]. The authors elude to the alteration in gut microbial composition causing increased susceptibility to inflammation due to a reduction in T-reg cells, which is commonly reported in cases of PTB [112]. Whilst these studies provide some support for a potential role for gut microbial inflammation in mediating PTB risk, there is clearly the need for more investigation in this area [113, 114].

## Manipulation of vaginal microbiota to modify PTB risk

Given that substantial evidence supports a link between specific vaginal microbiota composition and increased PTB risk, it is not surprising that numerous studies have examined the efficacy of antibiotics for the treatment and prevention PTB. These have almost entirely been targeted towards pregnant women with bacterial vaginosis (BV), and the results of these trials have proven highly inconsistent [115, 116]. In part, this is due to heterogeneity between trial designs (e.g. differences in the timing of treatment, types of antibiotic used, administration routes, patient cohorts selected and erroneous indications) [117, 118]. In the largest such trial to date, the double-blinded PREMEVA trial of oral clindamycin in early pregnancy [119] screened 85,000 women for BV with 1904 BV-positive participants (out of 5630 diagnosed) with low background risk for PTB assigned to the treatment arm and 956 participants assigned placebo consistent with the pre-trial defined 2:1 treatment:placebo ratio. No difference in the primary outcome of the composite of late miscarriage (16–21 weeks) or spontaneous very PTB (22–32 weeks) was detected between the two groups. Aspects of the PREMEVA trial design have come under scrutiny [117] [120], and there remain some experts who believe that a randomised, placebo-controlled trial of screening and treating BV in women with a history of PTB is still warranted [116]. However, there are a number of issues that need to be carefully considered in any such future trial. Treatment of women with existing BV implies that associated ascending infection or inflammatory pathway activation may have already commenced. Secondly, the use of some antibiotics (e.g. metronidazole) may cause lysing of bacteria and release of endotoxins [121], which are strong activators of inflammation in gestational tissues and therefore may potentiate an inflammatory phenotype [122–124]. Certain antibiotic treatments may be particularly effective against commensal species such as *Lactobacillus*, but not against pathogens associated with BV leading to inadvertent enrichment of these species in the vaginal niche [52, 125, 126]. Such trials would also need to be conducted with careful consideration of antibiotic resistance genes commonly found within BV-associated bacteria [127, 128].

The failure of antibiotic therapy to improve PTB in the context of abnormal vaginal flora, previous PTB or positive fetal fibronectin status has led to interest in the positive modulation of the vaginal microbiota using of live bio-therapeutic products (LBPs, live microorganisms, administered for the prevention or treatment of human disease) or probiotics (non-pathogenic microorganisms of human origin, given to provide health benefits). Probiotics may be given orally or less commonly vaginally. A systematic review of generally oral probiotic use in pregnancy found no evidence of any effect upon the PTB rate [129]. An observational study in Norway however found that probiotic milk consumption in early, but not mid to late pregnancy associates with a lower risk of PTB [130]. Two recently reported randomised controlled studies have found that oral probiotics, containing *Lactobacillus rhamnosus* GR-1 and *Lactobacillus reuteri* RC-14, do not modify the vaginal microbiota in pregnancy [131] [132].

An alternative approach is direct vaginal administration of *Lactobacillus* LBP*. L. crispatus* has been shown to inhibit growth of *Streptococcus agalactiae* (group B streptoccocus) in vitro. In non-pregnant women with a history of recurrent urinary tract infection, vaginal administration of *L. crispatus* following antibiotic treatment showed effective vaginal colonisation and a reduction in urinary tract infection recurrence [133]. Vaginal administration of both *L. acidophilus* and *L. rhamnosus* combined and *L. crispatus* has been shown a significantly reduced risk of recurrence in BV [134, 135]. Trials in pregnancy are currently in progress, but given the evidence for its protective effects, vaginal administration of suitable strains of *L. crispatus* represents a promising approach for beneficial modulation of the vaginal microbiota in pregnancy.

## Conclusions

The introduction of high-throughput DNA sequencing techniques into microbiological research has confirmed what was previously known from culture-based classical microbiology and greatly expanded our ability to study communities of microorganisms. Whilst it is doubtful that a physiologically essential placental microbiome exists, it remains possible that some cases of preterm labour may be due to haematogenous spread of organisms to the placenta and uterus. Studying the role of the gut microbiota in pregnancy and the potential for gut microbial inflammation to be linked to PTB risk will be challenging, but could potentially be paradigm changing. The strongest evidence for the role of the microbiome in preterm birth relates to the vaginal microbiota. But there remain significant geographical and ethnic differences, the underlying mechanisms of which are yet unknown and whose elucidation will provide valuable insights into the mechanisms of preterm birth in different ethnic groups and communities. The most consistent finding across almost all studies is the benefit of a vaginal microbiota characterised by *Lactobacillus crispatus*. This opens the door to modulation through the use of live bio-therapeutic products.
